# Assessment of thermotactile and vibrotactile thresholds for detecting sensorineural components of the hand–arm vibration syndrome (HAVS)

**DOI:** 10.1007/s00420-017-1259-2

**Published:** 2017-09-16

**Authors:** Ying Ye, Michael J. Griffin

**Affiliations:** Human Factors Research Unit, Institute of Sound and Vibration Research, University of Southampton, Southampton, England SO17 1BJ UK

**Keywords:** Vibrotactile perception thresholds, Thermotactile thresholds, Hand-transmitted vibration, Hand–arm vibration syndrome, Sensorineural dysfunction

## Abstract

**Background:**

Thermotactile thresholds and vibrotactile thresholds are measured to assist the diagnosis of the sensorineural component of the hand–arm vibration syndrome (HAVS).

**Objectives:**

This study investigates whether thermotactile and vibrotactile thresholds distinguish between fingers with and without numbness and tingling.

**Methods:**

In 60 males reporting symptoms of the hand–arm vibration syndrome, thermotactile thresholds for detecting hot and cold temperatures and vibrotactile thresholds at 31.5 and 125 Hz were measured on the index and little fingers of both hands.

**Results:**

In fingers reported to suffer numbness or tingling, hot thresholds increased, cold thresholds decreased, and vibrotactile thresholds at both 31.5 and 125 Hz increased. With sensorineural symptoms on all three phalanges (i.e. numbness or tingling scores of 6), both thermotactile thresholds and both vibrotactile thresholds had sensitivities greater than 80% and specificities around 90%, with areas under the receiver operating characteristic curves around 0.9. There were correlations between all four thresholds, but cold thresholds had greater sensitivity and greater specificity on fingers with numbness or tingling on only the distal phalanx (i.e. numbness or tingling scores of 1) suggesting cold thresholds provide better indications of early sensorineural disorder.

**Conclusions:**

Thermotactile thresholds and vibrotactile thresholds can provide useful indications of sensorineural function in patients reporting symptoms of the sensorineural component of HAVS.

## Introduction

Prolonged occupational exposure to hand-transmitted vibration has been associated with disorders in the vascular, neurological, and musculoskeletal structures of the human hand–arm system, collectively called the ‘hand–arm vibration syndrome’ (HAVS) (Griffin 1990). Vibration-induced neuropathy in the hand often manifested as reduced sensitivity, numbness, pain, tingling, or clumsiness in hand movements can reduce work ability and the quality of life (Anonymous 1995). It has been suggested that peripheral sensorineural symptoms may cause more discomfort and disability than vibration-induced vascular disorders, such as white finger, since this latter is episodic (usually triggered by exposure to cold) while sensory disturbances can be persistent and may interfere with life activities including sleep (Lundborg et al. 1990).

The hand–arm vibration syndrome is currently staged for severity according to the Stockholm Workshop scales for vascular disorders (Gemne et al. 1987) and sensorineural disorders (Brammer et al. 1987). These staging systems compound a mixture of signs and symptoms. In respect of sensorineural disorders, it is not clear what type of numbness or tingling is required or how reduced sensory perception, reduced tactile discrimination, or reduced manipulative dexterity should be measured, or what degree of reduction is required for a positive diagnosis (Griffin 2008). Consequently, there is no gold standard test to diagnose any stage of the sensorineural component of HAVS and many alternative tests have been suggested. In the UK, thermal thresholds and vibrotactile thresholds are recommended for assessing changes in sensorineural function associated with exposures to hand-transmitted vibration (Lindsell and Griffin 1998).

The perception of temperature is mediated by hot and cold receptors via unmyelinated C-fibres and thin myelinated A-δ fibres, respectively (Dyck et al. 1993). It has been reported that these nerve fibres can be damaged by occupational exposure to hand-transmitted vibration (e.g. Hirosawa et al. 1992). Currently, there is no internationally standardised method for the measurement and evaluation of thermotactile thresholds, although recommendations for thermal testing procedures have been provided by national academies, scientific associations, and research networks (Lindsell and Griffin 1998; Shy et al. 2003; Chong and Cros 2004; Rolke et al. 2006).

The perception of vibration is mediated by various mechanoreceptors via large-diameter myelinated A-α afferent nerve fibres (Burgess and Perl 1973). Using suitable apparatus and appropriate frequencies of vibration, the perception of vibration mediated by two different mechanoreceptors (Meissner’s and Pacinian corpuscles) can be determined. Various studies have concluded that the sensitivity of mechanoreceptors is reduced by occupational exposure to hand-transmitted vibration (Lundström et al. 1999; Bovenzi et al. 2011; Poole et al. 2016). The measurement of vibrotactile thresholds has been standardised in ISO 13091-1 (2001), and normative thresholds from a few studies have been suggested (ISO 13091-2 2003).

Both thermotactile thresholds and vibrotactile thresholds have been used to assist the diagnosis of disorders in patients reporting sensorineural symptoms of occupational origin (e.g. exposure to hand-transmitted vibration, industrial solvents, acrylamide) and non-occupational origin (e.g. diabetes, uraemia, and toxic-, infectious- or immune-associated neuropathies) (Zaslansky and Yarnitsky 1998; Shy et al. 2003; Chong and Cros 2004). The thresholds can be determined non-invasively using psychophysical methods to assess the integrity of the neuroanatomic pathway between the peripheral receptors and the sensory cortex. From a comparison of four thresholds (i.e. hot, cold, Meissner’s, and Pacinian), it is possible to identify whether any deterioration in perception is concentrated in one or more sensory unit or in one or more neural pathway.

For a group of males reporting symptoms of the hand–arm vibration syndrome, this paper compares thermotactile and vibrotactile thresholds between fingers with symptoms of numbness or tingling and fingers without symptoms of numbness or tingling. The tests were performed in accord with the HSE recommended procedure (Lindsell and Griffin 1998). It was hypothesised that there would be increases in vibrotactile thresholds at 31.5 and 125 Hz and increases in hot thresholds but decreases in cold thresholds in fingers reported to suffer numbness and tingling. It was further hypothesised that there would be positive correlations between the hot thresholds and vibrotactile thresholds and negative correlations between cold thresholds and vibrotactile thresholds, indicating that fingers more affected according to one test tend to be more affected according to another test.

## Methods

### Apparatus

Thermotactile thresholds (*HVLab* thermal aesthesiometer, University of Southampton).

An *HVLab* thermal aesthesiometer was used to measure thermotactile thresholds (hot and cold thresholds) via the method of limits (see Table 1). Thresholds were measured on the distal phalanx of the index and little fingers of the right and left hands.

**Table 1:** Parameters of the *HVLab* thermal aesthesiometer and *HVLab* vibrotactile perception meter

Parameter	Condition
Thermotactile thresholds	
Contact area	Circular, 5.5 cm diameter
Contact surface	Smooth and planar
Psychophysical method	Method of limits
Number of judgements	Six hot or cold
Reference temperature	32.5 °C
Rate of change in temperature	1 °C/s
Vibrotactile thresholds	
Probe diameter	6 mm
Probe surround gap	2 mm
Contactor surface	Smooth and planar
Psychophysical method	von Békésy
Number of judgements	At least six peaks and troughs
Rate of change in stimulus	3 dB/s
Push force	2 N

Subjects placed their fingertips so that the centre of the distal phalanx coincided with the centre of the applicator surface. They were instructed to apply a constant finger force of 2 N to the applicator surface, which they monitored using a digital scale located below the applicator. The temperature of the applicator increased or decreased from a reference temperature of 32.5 °C at a rate of 1 °C/s. Subjects were instructed to press the response button as soon as they perceived a change in temperature (i.e. increase or decrease in temperature). The temperature of the applicator then returned to the reference temperature and was held at 32.5 °C for a random interval before the temperature increased or decreased again.

Six hot and six cold thresholds were determined. For both thresholds, the mean was calculated from the last four judgements. The temperature difference between the hot threshold and the cold threshold, known as the thermal neutral zone, was also calculated on all four fingers.

Vibrotactile thresholds (*HVLab* vibrotactile perception meter, University of Southampton).

An *HVLab* vibrotactile perception meter was used to measure vibrotactile thresholds (thresholds at 31.5 and 125 Hz) via the von Békésy method in a manner compliant with the methods in ISO 13901-1 (2001) (see Table 1). Thresholds were measured on the distal phalanx of the index and little fingers of the right and left hands.

Subjects were instructed to place their fingertip so that the centre of the distal phalanx coincided with the centre of the probe of the applicator. The magnitude of vibration on the applicator increased from zero at the start of the test. Subjects were instructed to press and hold the response button down as soon as they perceived a vibration sensation and to release the response button as soon as they did not perceive the vibration.

Measurements were taken for a minimum of six reversals over at least 45 s, and the mean was calculated from all the peaks and troughs with the exception of the first peak and first trough.

### Subjects

Sixty male patients referred to the Human Factors Research Unit (University of Southampton) for Tier 5 testing for the hand–arm vibration syndrome agreed to participate in the study (Lindsell and Griffin 1998). This study reports findings from all 60 successive participants (i.e. there were no exclusions). The patients were medicolegal referrals and employer referrals.

The tiered system of health surveillance currently used in the UK has five stages (short questionnaire prior to work, annual questionnaire surveillance, HAVS assessment by an occupational nurse, diagnosis by occupational physician, and objective testing for signs of HAVS) (Health and Safety Executive 2005).

All 60 patients participating in the study had a history of smoking, but eight patients were not currently smoking. Fifty-three patients reported regular drinking of alcohol, five reported long-term medications, 12 had been exposed to chemicals at the workplace, and four had injury or surgery of their hands. Only one patient had noticed a change in skin quality (rough and dry).

They had their thermotactile thresholds and vibrotactile thresholds measured as part of their assessment. The subjects were in the clinic at a constant ambient temperature of 21 ± 1 °C, with 40–45% humidity and no noticeable air flow, for more than 1 h before the tests commenced.

All patients were right handed and with a history of occupational use of hand-held vibratory tools: a mean exposure of 23 years (SD: 6.8, range 5–45 years). They had used a wide range of vibratory tools in various jobs (e.g. gardener, maintenance worker, welder, fitter) with daily durations of exposure that varied between jobs. The subjects completed a health questionnaire and gave their written informed consent to participate in the study. The mean age of the patients was 52.9 years (SD: 12.4, range 30–70 years), their mean stature was 176.8 cm (SD: 5.7, range 165–190 cm), their mean weight was 83.1 kg (SD: 11.8, range 63–126 kg), and their mean body mass index (BMI) was 26.6 (SD: 5.7, range 19.6–38.8).

The subjects were requested to avoid consuming caffeine for 4 h and alcohol for 12 h prior to the testing. The study was approved by the Ethics Committee of the Faculty of Engineering and the Environment (Application number 10704).

### Procedure

Initially, patients were questioned about their occupational history, social and medical history, and symptoms (including any finger whiteness, numbness, tingling, and muscular problems). The locations of any finger blanching, numbness, or tingling were mapped using the scoring system (Griffin 1990, 2008). All decisions to include or exclude a symptom were made when identifying symptoms during interview prior to testing thresholds and therefore prior to data analysis.

The following tests were then performed in sequence for Tier 5 HAVS assessment: Purdue pegboard, grip strength, thermotactile thresholds, vibrotactile thresholds, finger rewarming, and finger systolic blood pressures. All tests were obtained using a computer with professional diagnostic software. The results were calculated, and diagnostic criteria were applied automatically within the software to minimise any bias introduced by the experimenter. The associations between vascular symptoms and signs in these patients have been reported elsewhere (Ye and Griffin 2016a).

### Statistical methods

Data analysis was performed using the software package SPSS (version 22.0). The data were summarised with the median as a measure of central tendency and the inter-quartile range as a measure of dispersion. Nonparametric tests were employed to analyse the data, which were not normally distributed. The Wilcoxon matched-pairs signed ranks test was used to investigate differences in finger skin temperatures before the measurement of thermotactile thresholds and vibrotactile thresholds. The Mann–Whitney *U* test was used to investigate differences between groups: fingers with and without symptoms of finger numbness or tingling. The Spearman rank correlation coefficient was used to investigate associations between thermotactile thresholds (hot and cold) and vibrotactile thresholds (at 31.5 and 125 Hz). The diagnostic criteria used in the study were: (1) hot thresholds greater than 45 °C and cold thresholds lower than 22 °C and (2) vibrotactile thresholds greater than 0.3 m/s^2^ r.m.s. at 31.5 Hz and greater than 0.7 m/s^2^ r.m.s. at 125 Hz. Receiver operating characteristic (ROC) analysis showed the effects of varying these criteria. The diagnostic accuracies of thermotactile thresholds and vibrotactile thresholds were investigated by calculating the areas under the ROC curves (AUC). The diagnostic criteria used in the study are shown in Table 2. The ‘possible dysfunction’ and ‘definite dysfunction’ (i.e. cut-off) values are one standard deviation and 2 standard deviations greater than mean values for healthy subjects reported previously (see Lindsell and Griffin 1998, 2002). The normative data of Lindsell and Griffin (2002) were obtained from 237 subjects in ten studies with 80 white collar workers and 157 blue collar workers within the UK. All subjects were male and of working age. No subjects reported symptoms of blanching, numbness, or tingling. No other medical conditions were reported.

**Table 2:** Diagnostic criteria for hot and cold thresholds and vibrotactile thresholds at 31.5 and 125 Hz

Test	Possible dysfunction	Definite dysfunction
Hot threshold (°C)	>45	>48.5
Cold threshold (°C)	<22	<19
31.5 Hz threshold (m/s^2^, r.m.s.)	>0.3	>0.4
125 Hz threshold (m/s^2^, r.m.s.)	>0.7	>1.0

Possible dysfunction is identified when the measured threshold differs from thresholds measured in a reference group of males without symptoms by more than the mean plus one standard deviation, and definite dysfunction is assumed when the difference is greater than the mean plus two standard deviations (Lindsell and Griffin 1998, 2002)

The criterion for statistical significance was *p* < 0.05. The reported *p* values have been adjusted for multiple comparisons.

## Results

The four tested fingers (index and little fingers on right and left hands) of the 60 patients reporting symptoms of the hand–arm vibration syndrome were categorised into two groups depending on the symptoms reported: Group A—fingers with sensorineural symptoms (i.e. fingers reported to have numbness or tingling) and Group B—fingers without sensorineural symptoms (i.e. fingers reported to be without numbness and without tingling). Temporary numbness and tingling can occur without a neurological disorder (e.g. as a normal response to vibration and other factors), so temporary numbness or tingling after exposure to hand-transmitted vibration, after gripping, or only associated with cold was excluded. Reports of numbness and tingling were not symmetrical and the areas affected differed between numbness and tingling. A ‘sensorineural score’ was calculated as the greatest of the score reported for numbness and the score reported for tingling, with a score of 1 for symptoms on the distal phalanx, 2 for the middle phalanx, and 3 for the proximal phalanx (Griffin 1990). For example, if a finger had a ‘numbness score’ of 6 (numbness on all three phalanges) and a ‘tingling score’ of 1 (tingling on the distal phalanx), the ‘sensorineural score’ was 6. The symptoms differed between fingers, so the number of fingers in Group A and Group B varied between fingers. Of the 240 fingers tested, 154 fingers were reported to have sensorineural symptoms (144 fingers had numbness, 131 fingers had tingling, and 154 had either numbness or tingling or both numbness and tingling) and 136 fingers were reported to have vascular symptoms (i.e. finger blanching provoked by cold conditions).

Statistical analysis has been performed on sensorineural scores reported between fingers within subjects; there was no significant correlation between symptoms reported on different fingers within subjects (*p* = 0.108–0.377, Spearman).

The finger skin temperatures measured prior to measuring thermotactile thresholds did not differ from the temperatures measured prior to measuring vibrotactile thresholds on either the right or left hands (*p* = 0.156–0.387).

There were lower baseline finger skin temperatures on fingers with sensorineural symptoms (Group A, median: 27.2 °C, range 23.8–32.4 °C) compared with fingers without symptoms (Group B, median: 28.8 °C, range 25.1–34.1 °C) (*p* = 0.006–0.027).

### Thermotactile thresholds

The medians and inter-quartile ranges of hot thresholds and cold thresholds on fingers in Group A and Group B are shown in Table 3. The neutral zones are also shown in Table 3.

**Table 3:** Medians (and inter-quartile ranges) of hot thresholds, cold thresholds, neutral zones, and vibrotactile thresholds at 31.5 and 125 Hz on the distal phalanges of fingers with sensorineural symptoms (Group A) and fingers without sensorineural symptoms (Group B)

	154 fingers with any sensorineural symptom (Group A)	86 fingers without any sensorineural symptom (Group B)
Hot thresholds (°C)	48.2 (45.7–50.9)**	41.4 (39.2–43.0)
Cold thresholds (°C)	16.8 (14.2–21.3)**	25.8 (24.3–28.0)
Neutral zone (°C)	30.6 (24.4–32.7)**	15.3 (13.8–19.7)
Thresholds, 31.5 Hz (m/s^2^ r.m.s.)	0.41 (0.34–0.59)**	0.17 (0.14–0.25)
Thresholds, 125 Hz (m/s^2^ r.m.s.)	1.19 (0.86–1.72)**	0.35 (0.28–0.61)

* *p* < 0.01, ** *p* < 0.001: significant increase in hot threshold or decrease in cold thresholds on fingers with sensorineural symptoms compared to fingers without sensorineural symptoms (Mann–Whitney *U* test)

There were no significant differences in either hot or cold thresholds between the four fingers (i.e. four locations) in either Group A or Group B (*p* = 0.132–0.476). The hot thresholds were higher in Group A than in Group B (*p* < 0.01). The cold thresholds were lower in Group A than in Group B (*p* < 0.001). The thermal neutral zone was greater in fingers with sensorineural symptoms than in fingers without sensorineural symptoms (*p* < 0.001). The mean difference (with 95% confidence interval) between fingers with and without sensorineural symptoms for hot thresholds was 6.91 (CI of 5.81–8.01) with an effect size of 1.66 (CI of 1.35–1.96). The mean differences (with 95% confident intervals) between fingers with and without sensorineural symptoms for cold thresholds and for the neutral zone were 8.63 (CI of 7.87–9.39) and 15.52 (CI of 13.98–17.06), with effect sizes of 2.59 (CI of 2.23–2.93) and 2.68 (CI of 2.31–3.02), respectively.

On fingers with sensorineural symptoms (i.e. Group A), a greater change in threshold for the detection of hot and cold temperatures was found on fingers with a sensorineural score of 6 than on fingers with a sensorineural score of 1 or 3 (*p* < 0.01). There were no significant differences in either hot thresholds or cold thresholds between fingers with sensorineural scores of 1 and 3 (*p* = 0.217–0.829).

The finger skin temperatures measured prior to the measurement of thermotactile thresholds were not correlated with the hot or cold thresholds at any of the four locations in either Group A or Group B (*p* = 0.128–0.635).

The sensitivities and specificities of the thermotactile thresholds to distinguish between fingers with and without sensorineural symptoms were 81 and 92% for hot thresholds, 87 and 94% for cold thresholds, and 77 and 88% for the neutral zone. The area under the receiver operating characteristic curve and 95% confidence intervals (CI) were 0.89 (CI of 0.81–0.97) for hot thresholds, 0.96 (CI of 0.92–1.00) for cold thresholds, and 0.81 (CI of 0.75–0.87) for the neutral zone. The diagnostic odds ratio (DOR) and 95% confidence intervals for hot thresholds, cold thresholds, and the neutral zone were 47.0 (CI of 20–112), 107 (CI of 38–296) and 25 (CI of 12–53).

**Fig. 1:**
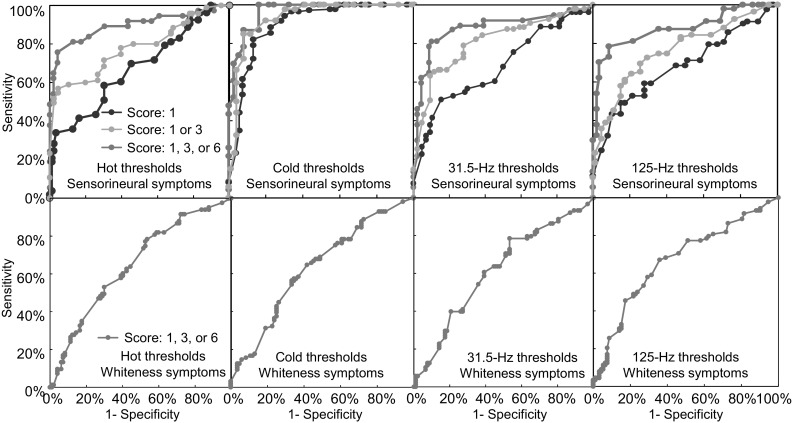
Receiver operating characteristics (ROC) curves for sensorineural symptoms (top) and finger whiteness (bottom) calculated for hot and cold thresholds and vibrotactile thresholds at 31.5 and 125 Hz. The ROC curves are shown for sensorineural scores of 1, 3, and 6 and for finger whiteness scores of 1, 3, or 6. Data from 60 subjects

The ROC curves for sensorineural symptoms calculated for hot and cold thresholds and vibrotactile thresholds at 31.5 and 125 Hz are shown in the upper part of Fig. 1. The ROC curves are shown separately according to the severity of the reported symptoms. The corresponding ROC curves for any symptoms of finger blanching (scores of 1, 3, or 6) calculated for hot and cold thresholds and vibrotactile thresholds at 31.5 and 125 Hz are shown in the lower part of Fig. 1.

### Vibrotactile thresholds

The medians and inter-quartile ranges of vibrotactile thresholds measured on the index and little fingers of the right and left hands at 31.5 and 125 Hz in Group A and Group B are shown in Table 3.

There were no significant differences in vibrotactile thresholds at either 31.5 or 125 Hz between the four fingers (i.e. four locations) in either Group A or Group B (*p* = 0.212–0.539). The vibrotactile thresholds at 31.5 and 125 Hz were significantly higher in Group A than in Group B (*p* < 0.001). The mean differences (with 95% confident intervals) between fingers with and without sensorineural symptoms for vibrotactile thresholds at 31.5 and 125 Hz were 0.31 (CI of 0.26–0.36) and 0.80 (CI of 0.62–0.98), with effect sizes of 1.73 (CI of 1.42–2.03) and 1.16 (CI of 0.87–1.44), respectively.

On fingers with sensorineural symptoms (i.e. in Group A), vibrotactile thresholds at both 31.5 and 125 Hz were greater on fingers with sensorineural scores of 3 and 6 than on fingers with a sensorineural score of 1 (*p* < 0.05). There were no significant differences in thresholds at 31.5 and 125 Hz between fingers with sensorineural scores of 3 and 6 (*p* = 0.092–0.418).

The finger skin temperatures prior to measuring the vibrotactile thresholds were not correlated with vibrotactile thresholds at 31.5 Hz at any of the four locations in either Group A or Group B (*p* = 0.148–0.335). However, there was a negative correlation between finger skin temperatures and vibrotactile thresholds at 125 Hz at all four locations in Group B (*p* < 0.05), but not in Group A (*p* = 0.418–0.571).

The sensitivities and specificities of the vibrotactile thresholds to distinguish between fingers with and without sensorineural symptoms were 80 and 91% for thresholds at 31.5 Hz and 82 and 91% for thresholds at 125 Hz. The AUC and 95% confidence intervals were 0.90 (CI of 0.84–0.96) for 31.5-Hz thresholds and 0.91 (CI of 0.85–0.97) for 125-Hz thresholds (see Fig. 1). The diagnostic odds ratios and 95% confidence intervals for vibrotactile thresholds at 31.5 and 125 Hz were 39 (CI of 17–89) and 44 (CI of 19–101), respectively.

### Associations between thermotactile thresholds and vibrotactile thresholds

**Fig. 2:**
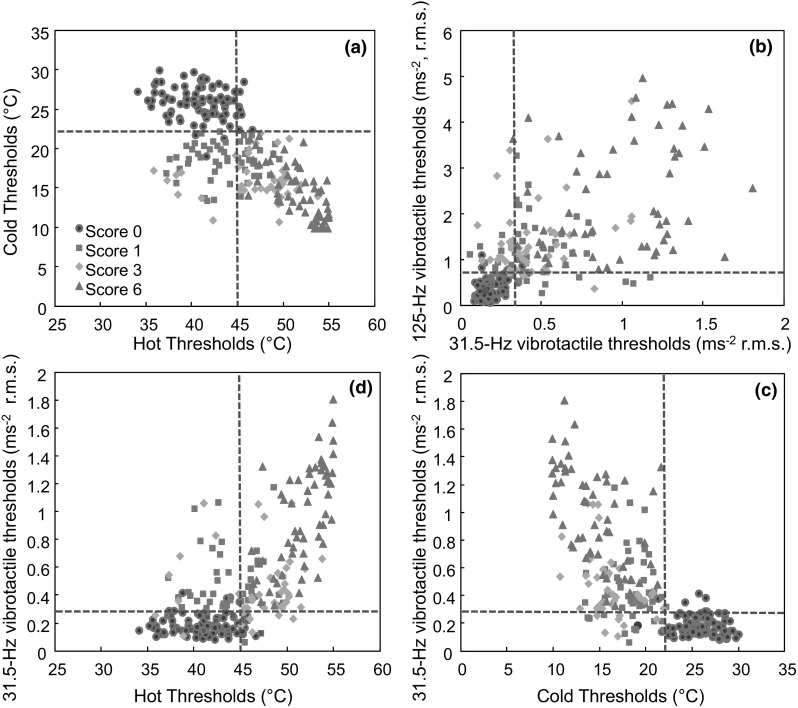
Associations between **a** hot and cold thresholds, **b** vibrotactile thresholds at 31.5 and 125 Hz, **c** vibrotactile thresholds at 31.5 Hz and cold thresholds and **d** vibrotactile thresholds at 31.5 and hot thresholds in fingers with sensorineural scores of 0, 1, 3, and 6. Broken lines indicate diagnostic criteria for possible dysfunction (see Table 2). Data from 60 subjects

For fingers with sensorineural scores of 0, 1, 3, and 6, examples of associations between (a) hot and cold thresholds, (b) vibrotactile thresholds at 31.5 and 125 Hz, (c) 31.5-Hz thresholds and cold thresholds and (d) 31.5-Hz thresholds and hot thresholds are shown in Fig. 2.

In Group A (with symptoms), there was a negative correlation between hot and cold thresholds on fingers with a sensorineural score of 6 (*p* = 0.017–0.031), but not on fingers with sensorineural scores of 1 or 3 (*p* = 0.065–0.327). In Group B (without symptoms), there was no statistically significant correlation between hot and cold thresholds on any of the four fingers (*p* = 0.311–0.623).

In Group A, there was a positive correlation between vibrotactile thresholds at 31.5 and 125 Hz on fingers with a sensorineural score of 6 (*p* < 0.01), but not on fingers with sensorineural scores of 1 and 3 (*p* = 0.094–0.169). In Group B, there was a positive correlation between vibrotactile thresholds at 31.5 and 125 Hz on all four fingers (*p* < 0.05).

In Group A, there were positive correlations between hot thresholds and vibrotactile thresholds (at both 31.5 and 125 Hz) and negative correlations between cold thresholds and vibrotactile thresholds (at both 31.5 and 125 Hz) on fingers with a sensorineural score of 6 (*p* < 0.05), except between cold thresholds and vibrotactile thresholds at 125 Hz (*p* = 0.064–0.103). On fingers with a sensorineural score of 1 or 3, there were no statistically significant correlations between thermotactile thresholds (hot or cold) and vibrotactile thresholds (at 31.5 or 125 Hz) (*p* > 0.1). In Group B, the thermotactile thresholds (for hot and cold) and vibrotactile thresholds (at 31.5 and 125 Hz) were not significantly correlated with each other on any of the four fingers (*p* = 0.196–0.350).

### Associations between thresholds and HAVS symptoms (numbness, tingling, and whiteness)

The sensitivity, the specificity, and the AUC were calculated for three reported HAVS symptoms (numbness, tingling, and whiteness) with four thresholds (hot and cold thresholds, vibrotactile thresholds at 31.5 and 125 Hz). The findings summarised in Table 4 for the total of 240 fingers were obtained where a symptom corresponded to a score of 1 or greater.

**Table 4:** Sensitivity, specificity, and area under the ROC curve (AUC) calculated for thermotactile and vibrotactile thresholds using symptoms of numbness, tingling, and whiteness

Measure	Numbness			Tingling			Whiteness		
	Sen. (%)	Spec. (%)	AUC	Sen. (%)	Spec. (%)	AUC	Sen. (%)	Spec. (%)	AUC
Hot thresholds	80	91	0.88	76	83	0.82	53	65	0.60
Cold thresholds	85	92	0.94	82	88	0.87	50	63	0.59
Vibrotactile thresholds at 31.5 Hz	77	88	0.85	73	85	0.82	49	64	0.58
Vibrotactile thresholds at 125 Hz	79	89	0.86	72	83	0.82	52	68	0.64

Statistical analysis was also performed on thresholds between fingers with and without any blanching. There were significantly lower thresholds in fingers with blanching (*p* < 0.05), but no difference in any of the four thresholds between fingers with blanching scores of 1, 3, and 6 (*p* = 0.079–0.331).

## Discussion

### Thermotactile thresholds

In the present study, when using the tests and criteria currently employed in the UK, thermal perception thresholds provided useful indications of whether fingers had sensorineural symptoms: 81% sensitivity and 92% specificity for hot thresholds and 87% sensitivity and 94% specificity for cold thresholds. Thermal dysfunction was observed in digits innervated by both the median nerve (index finger) and the ulnar nerve (little finger). The findings are consistent with previous studies of thermal dysfunction in the fingers of various groups of vibration-exposed workers, including users of chain saws, hand-held grinders, and hammer drills (e.g. Virokannas and Virokannas 1995; Lindsell and Griffin 1999; Toibana et al. 2000; Nilsson and Lundström 2001; Sakakibara et al. 2002; Bovenzi et al. 2011).

Although conventional electro-neurophysiological methods cannot measure the anatomical integrity of small-calibre nerve fibres, their functional capacity can be assessed using a thermal aesthesiometer (Magda et al. 2002; Nilsson et al. 2008). However, there is no international standard for the measurement and evaluation of thermal thresholds and only a few studies have provided normative data for thermotactile thresholds in a healthy population (e.g. Nilsson et al. 2008; Seah and Griffin 2008). In the current study, hot and cold thresholds on fingers without sensorineural symptoms were similar to those reported previously for the same instrument and methodology (i.e. the same contact conditions, psychophysical method, reference temperature, and rate of change in temperature) (Lindsell and Griffin 1999; Seah and Griffin 2008; Bovenzi et al. 2011).

In the ROC curves, the greater the central portion of a curve moves upwards and to the left, the greater a measure distinguishes between fingers with and without sensorineural symptoms. In the ROC curve analysis, AUCs of 0.8 and 0.65 are described as having, respectively, ‘good’ and ‘fair’ discriminative ability (Weinstein and Fineberg 1980). For the hot and cold thresholds, the AUC was 0.89 and 0.96, respectively, in the current study, indicating the thermotactile thresholds provided ‘good’ indications of whether a finger had sensorineural symptoms.

Some studies have concluded that the neutral zone between hot and cold thresholds was a sensitive indicator of nerve damage (Hirosawa et al. 1983; Ekenvall et al. 1986), while others have concluded that cold thresholds were more useful than hot thresholds in the detection of vibration-induced neuropathy (Ekenvall et al. 1986; Virokannas and Virokannas 1995). Nilsson et al. (2008) reported reduced perception to cold but no significant changes in hot thresholds among young adults exposed to hand-transmitted vibration. In the present study, although greater sensitivity and greater specificity were obtained with cold thresholds, the difference was small and the patients appear to have been damaged similarly in their hot and cold receptors or neurological pathways. In patients with severe neurological injury, both the hot and the cold thresholds may be abnormal, whereas in patients with less injury one thermotactile channel may be affected than the other. Differences in the method of measuring thermal thresholds may also affect which channel appears to be most affected, as thermotactile thresholds depend on many factors including the starting temperature, the area of contact and the contact location (Hilz et al. 1995; Ruffell and Griffin 1995; Seah and Griffin 2010).

### Vibrotactile thresholds

Vibrotactile thresholds at 31.5 and 125 Hz reflect the responses of two different mechanoreceptors in the skin and their afferent fibres (ISO 13091-1 2001). This study suggests vibrotactile thresholds can be powerful indicators of sensorineural symptoms of HAVS. They had sensitivities around 80% and specificities around 90% with an AUC greater than 0.9. This is consistent with previous investigations, suggesting vibration perception thresholds can be used to assess sensory loss in the fingers of vibration-exposed workers with a history of sensorineural symptoms (e.g. Ekenvall et al. 1986, 1989; Virokannas and Virokannas 1995; Lundström et al. 1999; Bovenzi et al. 2011). Vibrotactile thresholds on fingers without sensorineural symptoms were consistent with those previously measured at other European test centres using the same measurement apparatus, psychophysical methods, and skin–stimulus contact conditions (Lindsell and Griffin 2003). Although clinical electrophysiology may be used to assess large fibre functions, vibrotactile thresholds have been reported to be more sensitive than conventional neurography (Rolke et al. 2006).

### Association between thermotactile thresholds and vibrotactile thresholds

On fingers with more severe symptoms (i.e. sensorineural score of 6), thermotactile thresholds were correlated with vibrotactile thresholds. On fingers with less extensive sensorineural symptoms (sensorineural scores of 1 and 3), the four thresholds (i.e. hot and cold thresholds and vibrotactile thresholds at 31.5 and 125 Hz) were not correlated with each other. This is partially consistent with Toibana et al. (2000) who found a significant correlation between thermal thresholds and pain thresholds but not between thermal thresholds and vibrotactile thresholds. The neural pathways for these four sensory systems differ: whereas the perception of vibration is mediated by the responses of Meissner’s and Pacinian corpuscles and large-diameter myelinated Aα afferent nerve fibres, the perception of warmth is mediated by thinly myelinated Aδ nerve fibres and the perception of coolness is mediated by unmyelinated C nerve fibres (Burgess and Perl 1973). Every nerve in the peripheral system has a specific function, so the signs and symptoms depend on the type of nerve affected. The functions of different peripheral receptors, neural pathways, and components of the sensory cortex are therefore evaluated by measuring the different thermotactile and vibrotactile thresholds. In some cases, a finger with a sensorineural score of 1 had reduced sensitivity to cold but had a normal hot threshold and normal vibrotactile thresholds. The absence of a statistically significant correlation between hot and cold thresholds on fingers with symptoms (numbness or tingling) reported on the distal and middle phalanges of a finger indicates the need for separate tests for hot and cold thresholds, rather than a single measure of the ‘neutral zone’.

Cold thresholds had greater sensitivity and specificity than either hot thresholds or vibrotactile thresholds in fingers with mild symptoms (with sensorineural score of 1), consistent with reduced sensitivity to cold reported among young adults exposed to hand-transmitted vibration (Nilsson et al. 2008). Some studies of patients with the hand–arm vibration syndrome have concluded that thresholds for the perception of hot and cold temperatures are impaired before vibrotactile thresholds (Ekenvall et al. 1989; Virokannas and Virokannas 1995; Bovenzi et al. 2011), consistent with alterations in the thin non-myelinated fibres in nerve tissues of rats after exposure to vibration (Lundborg et al. 1990).

The cross-sectional design of the present study did not allow the investigation of changes in the signs and symptoms of sensory function in fingers over time—this requires a longitudinal study of the relation between vibration exposure and sensory function. The study did not, therefore, seek to investigate the cause of the symptoms or signs and merely investigate differences in thresholds between fingers with and without sensorineural symptoms.

The study also found that thermotactile thresholds and vibrotactile thresholds were more impaired among patients with moderate and severe sensorineural symptoms, consistent with studies suggesting that thermotactile and vibrotactile thresholds can reflect the severity of nerve damage (Hirosawa et al. 1983; Toibana et al. 2000; Poole et al. 2016).

For all four thresholds, there was greater sensitivity and greater specificity for numbness than for tingling (Table 4), although patients with either numbness or tingling are likely to have impaired thermotactile and vibrotactile thresholds. Although the numbness and tingling symptoms were not identically distributed, the affected fingers were similar, so the study does establish which signs corroborate which symptoms.

Lower thresholds were found on fingers with blanching, but the sensitivity and specificity of thresholds for the detection of blanching were low (around 55%). On fingers with blanching, there were no significant differences in thresholds between fingers with different blanching scores. So neither thermotactile thresholds nor vibrotactile thresholds were useful indicators of the presence or the severity of vascular dysfunction—the small association between thresholds and blanching may arise because both are caused by exposure to hand-transmitted vibration. The findings are consistent with Ekenvall et al. (1989) who found no relation between vascular symptoms and the outcome of sensory testing. Whereas the chronic changes giving rise to finger blanching are not clearly associated with thresholds, acute reductions in finger blood flow caused by vibration are associated with thresholds for the perception of vibration (Ye and Griffin 2011, 2014, 2016b). The contradiction implies that the acute and chronic changes have different mechanisms, with the vascular impairment in vibration-exposed persons being associated with either a different form of neurological damage or some form of local structural damage causing the vascular phenomenon known as vibration-induced white finger.

The current study was conducted within patients referred for Tier 5 HAVS assessment. According to the tiered system used in UK, prior to the test, all patients will have been interviewed by an occupational physician who considered it likely the patient had HAVS, but recommended objective tests before confirming the decision. This means the patients were pre-selected by various occupational physicians, and all patients were likely to have at least one of the HAVS symptoms (numbness, tingling, or whiteness) on at least one finger. The sensitivities and specificities found in this study can be expected to be dependent on the population studied, including the extent to which patients had been exposed to hand-transmitted vibration, had relevant symptoms, or had other conditions that could affect either the symptoms or the signs of disorder. Although limitations to the testing time meant that only two fingers were tested on each hand, the finding of associations between the symptoms and signs on a finger should encourage the measurement of thresholds on all five fingers of both hands

Self-reported numbness and tingling scores can be overestimated or underestimated, but it is unlikely this was a major issue since the sensitivity and specificity show that thresholds were ‘good’ indicators of whether a finger had sensorineural symptoms. Furthermore, errors in reporting numbness and tingling would not have affected the relative performance of the four threshold tests. During interview with the patients, they were asked how often they experienced numbness and tingling (e.g. few times a year or few times a day), the duration of the symptoms (e.g. transient or permanent), and the conditions in which the symptoms occurred (e.g. during or after using vibrating tools, in cold condition). A single exposure to vibration may cause a temporary period of numbness or tingling that might be accompanied by impaired sensory perception, so it might be considered ‘normal’ for vibration-exposed workers to have intermittent numbness or tingling. Away from work, and when tested in a clinic, such temporary effects will have passed, so it seems reasonable to exclude them when judging the symptoms and when relating symptoms to signs. Temporary numbness or tingling was excluded in this study, but this does not imply that temporary effects are of no interest as they might be indicators of the likelihood of more permanent effects.

Impaired thermotactile or vibrotactile perception in a vibration-exposed worker is not a sufficient sign to diagnose the sensorineural component of the hand–arm vibration syndrome. Elevated thresholds in a finger should be considered in relation to a history of exposure to hand-transmitted vibration, symptoms in the same finger, and absence of alternative explanations of the signs and symptoms. Reduced sensitivity to temperature or vibration could imply damage to peripheral nerves caused by various conditions: traumatic injuries, infections, metabolic problems, diabetes mellitus or exposure to chemicals or toxins (Hughes 2002). With increased age, healthy men and women have been reported to have reduced sensitivity to both temperature (e.g. Bartlett et al. 1998; Lindsell and Griffin 2002) and vibration (Bartlett et al. 1998; Wild et al. 2001). The influences of general health, alcohol consumption, smoking, gender, profession, and age on thermotactile and vibrotactile thresholds merit greater attention.

## Conclusions

For the conditions and protocols applied in the present study, both thermotactile thresholds and vibrotactile thresholds differed between fingers with and without sensorineural symptoms. Cold threshold had greater sensitivity and greater specificity on fingers with numbness or tingling scores of 1, suggesting cold thresholds provide better indications of early sensorineural disorder.
